# Intensive Care Unit (ICU)-Related Post-traumatic Stress Disorder: A Literature Review

**DOI:** 10.7759/cureus.57049

**Published:** 2024-03-27

**Authors:** Ahmed M Abdelbaky, Mohamed S Eldelpshany

**Affiliations:** 1 Intensive Care Unit, Rashid Hospital - Dubai Health, Dubai, ARE

**Keywords:** risk factors, psychological consequences, post-traumatic stress disorder, intensive care unit, icu

## Abstract

An intensive care unit (ICU) is a challenging environment characterized by frequent incidences of stressors and traumatic situations. Therefore, both patients and caregivers are at high risk of developing psychological disorders such as post-traumatic stress disorder (PTSD), anxiety, and depression. ICU-related PTSD is a significant concern that remains under-recognized. This literature review examines the current state of knowledge regarding ICU-related PTSD, including its prevalence, risk factors, clinical manifestations, and potential interventions. Key findings suggest that a considerable proportion of ICU survivors develop PTSD symptoms, which can significantly impact their quality of life and recovery. The most common predictors investigated for PTSD in ICU survivors are age, gender, pre-illness psychopathy, length of stay in the ICU, delirium, and sedative agents. The treatment and prevention strategies of ICU-related PTSD include psychological therapies and pharmacological and non-pharmacological treatments. Psychological interventions, including cognitive-behavioral therapy and pharmacotherapy, have shown promise in mitigating PTSD symptoms in ICU survivors. However, further research is needed to better understand the mechanisms underlying ICU-related PTSD and to develop targeted interventions to prevent and manage this debilitating condition.

## Introduction and background

An intensive care unit (ICU) is an environment filled with a lot of stressors and traumatic events. Therefore, such an experience can be traumatizing for some patients as they sometimes have to confront their mortality [1]. Some studies have reported an ICU-related mortality of 16.9-29.6% [2,3]. This variation in ICU-related mortality is attributed to ICU location, setting, and frequency of patient visits. Due to the high incidence of end-stage diseases and mortality, the announcement of death and poor prognosis is common in these settings which can have a significant impact on the mental health of patients and health professionals in the ICU. Furthermore, this type of environment fulfills various risk factors reported by the Diagnostic and Statistical Manual of Mental Disorders, Fifth Edition (DSM-5) which include confronting death personally, death of people you are close to, or indirect exposure as witnessed by ICU professionals [4]. Some of the most common symptoms of PTSD include nightmares, memories of the trauma, hypervigilance, irritability, emotional withdrawal, poor concentration, and difficulty sleeping [5]. If these symptoms persist for more than one month after an ICU visit, then the condition is diagnosed as PTSD; otherwise, it is deemed an acute stress disorder [6].

PTSD is thought to occur in 25-30% of the people who have experienced a distressing event [7]. When patients are admitted to ICU, they face a highly stressful environment surrounded by medical equipment and unfamiliar faces. In such a scenario, some factors further exacerbate patients' situation such as delirium, sedation, confusion, and intubation. Furthermore, they make their communication difficult and even impossible [7]. Around 270,000 patients are admitted to the ICU every year, and approximately 27% of them develop PTSD. This condition can be attributed to various factors including immobility, pain, sleep disturbances, and sensory deprivation. The families of ICU patients are also at risk of developing PTSD [8]. Family members, particularly those involved in the main decision-making role for the relative admitted to the ICU, are also at a 33% risk of developing PTSD. The risk of developing PTSD among these family members can increase, especially if the patient passes away in the ICU or if there is insufficient information provided during end-of-life decision-making [9].

Events unfolding in an ICU setting can also have a significant impact on the mental status of health professionals [10]. PTSD has also a high risk of becoming a chronic condition and is associated with frequent psychiatric comorbidities such as anxiety, depression, addictions, and suicidal behavior [6]. Previously, several researchers have investigated ICU-related PTSD for the past 15 years. Despite these efforts, PTSD following an ICU-related traumatic experience is still an under-recognized area. Therefore, the present review aims to summarize the current understanding of ICU-related PTSD, exploring the key risk factors associated with its development, its impact on patient's lives, and potential avenues for prevention and treatment. This review is focused on synthesizing the existing literature and providing valuable insights that will not only aid healthcare providers in identifying at-risk patients but also pave the way for the development of targeted interventions.

## Review

Methodology

For this literature review, a search was undertaken in various key databases including PubMed, CINAHL Ultimate, and Web of Science. Furthermore, Google Scholar was also searched to find relevant studies on the topic. The search was carried out using keywords such as "intensive care unit," or "ICU," or "critically ill" and "PTSD," or "post-traumatic stress disorder." The details of search words are described in Appendix 1. The search was not limited by the duration or type of study. To identify relevant literature, original studies, review articles, and systemic reviews were also analyzed. Studies were included if they assessed various predictors of ICU-related PTSD, prevalence and outcomes of PTSD, screening for PTSD, and various management options for ICU-related PTSD.

Results and discussion

Predictors of ICU-Related PTSD

The most common predictors investigated for PTSD in ICU survivors are age, gender, pre-illness psychopathy, length of stay in the ICU, delirium, and sedative agents. Severe illness and long ICU stays are thought to be the most common risk factors in ICU-related PTSD. Battle et al., in their study that involved 198 patients admitted to the ICU, reported that 27% of patients were suffering from PTSD. Multivariable logistic regression revealed that younger age, pre-illness psychopathy, and a lower Apache score are risk factors for ICU-related PTSD [11]. However, Ratzer et al. reported that ICU length of stay and severity of illness have no association with PTSD in ICU survivors [12].

In their multicenter study, Unoki et al. reported that an unscheduled admission to the ICU was also a predictive factor in the more severe form of PTSD, depression, and anxiety in the patients [13]. The emergence of PTSD in the pediatric ICU was reported in a recent study. The study highlighted various risk factors for the development of PTSD in one out of every four children in the ICU. These factors include the onset of intensive care delirium, the use of benzodiazepines, and the development of PTSD in the parents. Some other factors such as acute child and parent stress, sepsis, and emergency admission were considered as potential risk factors, but they still require further investigation. Gender and age were not significantly associated with PTSD in the pediatric population [14].

Delirium is one of the most common behavioral manifestations of brain dysfunction in the ICU. It is actually a disturbance of consciousness, changes in cognition, and the development of a perceptual disturbance [15]. Delirium is also presented as one of the main predictors of ICU-related PTSD in various studies [11,14]. Delusional memories have been related to an increased risk of developing high severity of PTSD symptoms and PTSD in critically ill patients [15]. Amnesia in the early period of critical illness is also associated positively with the levels of PTSD-related symptoms [16].

The duration and type of ICU sedation are also related to the risk of post-ICU PTSD. The data regarding this risk factor is conflicting and non-confirmatory. Benzodiazepines are thought to play a role in this scenario. Additionally, lorazepam, opiates, and midazolam are also found to have links with post-ICU PTSD [15]. On the other hand, a comprehensive and large ICU-related PTSD study found no results between benzodiazepine, opiate dose, and delirium [17].

Prevalence and Outcomes of ICU-Related PTSD

The incidence of ICU-related PTSD varies in the published research based on the location of the study, follow-up duration, and number of visits to the ICU. A recent systematic review and meta-analysis indicated a pooled prevalence of 19.83% of PTSD after an ICU stay. They further revealed that PTSD may affect one person out of every five ICU survivors [18]. A prospective multicenter study of ICU survivors in the United Kingdom indicated that 18% of the patients had PTSD even one year after being discharged from the ICU [19]. The adverse outcomes of PTSD include various mental and psychological health issues. Depression and anxiety along with PTSD were reported in 32% and 38% of patients after being discharged from the ICU [19]. Another study reported a 6% prevalence of PTSD, while the prevalence of anxiety was 16.6%, and depression was found to be 28.1% [13].

The prevalence of PTSD in ICU professionals has also been investigated previously. The prevalence of PTSD in ICU professionals ranges from 3.3% to 24% [20,21]. Although all roles within the ICU are affected, nursing staff are particularly vulnerable to PTSD [22]. The high prevalence of ICU-related PTSD is known to impact the health-related quality of life. This was confirmed by the systematic review of Davydow et al. which reported that ICU-related PTSD was associated with significantly lower health-related quality of life [23]. Additionally, PTSD can also result in impaired daily functioning and decreased life course opportunities [8]. Trauma survivors also display cognitive impairment. This cognitive impairment is associated with poor quality of life, inability to return to work, and functional defects [24].

Screening and Assessments of ICU-Related PTSD

There is a variety of screening and assessment tools used for assessing the symptoms of ICU-related PTSD. Some of the commonly used tools are listed below.

Post-Traumatic Stress Symptoms Checklist-10 (PTSS-10): PTSS-10 is a valid screening tool for the assessments of PTSD-related symptoms in ICU survivors. There are two parts to this screening tool. Part A consists of four questions related to feelings and memories of the traumatic events in the ICU such as trouble breathing, panic attacks, anxiety, pain, and nightmares. These questions can be answered with no or yes. There are a total of 10 questions in the part B that are related to the ongoing stress symptoms. These questions can be answered in scores from 1 (never) to 7 (always). The total scores range from 10 to 70 points. If a patient's scores are above 34 in part B, then they will be indicative of the presence of clinically significant PTSD symptoms. These symptoms will be associated with a diagnosis of PTSD [25].

Hospital Anxiety and Depression Scale (HADS): HADS is a valid scale for both critically ill and general medical patients. This questionnaire consists of two subscales. These subscales measure the depression and anxiety of the patients. There are a total of seven items in each subscale scored from 0 to 3, resulting in a subscale score that ranges from 0 to 21. If the score is above 7 in a subscale, there will be clinically significant problems [25].

Impact of Event Scale-Revised (IES-R): IES-R is a reliable and valid tool. IES-R is also being used to measure the symptoms of PTSD. This instrument consists of a total of 22 items and three subscales. These subscales include hyperarousal, avoidance, and intrusion. The 22 items are scored on a range from 0 (not at all) to 4 (extremely). This tool allows a maximum of 88 points. IES-R has concurrent validity (r=0.80) and a high internal consistency (α=0.96) [26].

Clinician-Administered PTSD Scale for DSM-5 (CAPS-5): CAPS-5 is a structured interview that measures the severity and frequency of each symptom of PTSD concerning a single traumatic stressor over one month. This scale allows the trained interviewer to conclude a lifetime or current diagnosis corresponding to the DSM-5 of the American Psychiatric Association. This scale consists of 30 items [27].

Posttraumatic Stress Disorder Checklist for DSM-5 (PCL-5): PCL-5 is a self-report measure that measures the presence and severity of PTSD symptoms according to the standards outlined in the DSM-5. This scale includes 20 items that are rated on a 5-point Likert scale. The categories of responses are 1 (not at all) to 5 (extremely) regarding the traumatic experiences. The factors of the scale are avoidance, intrusions, reactivity and arousal, and negative changes in mood and cognition. PTSD diagnosis can be made if the score of each item is 2 or above 2 [28].

Prevention and Treatment Strategies for ICU-Related PTSD

The precise nature of the interventions needed to reduce the prevalence of ICU-related PTSD has not yet been defined. However, an earlier intervention can be more beneficial to lower the prevalence of symptoms of PTSD [29].

Non-pharmacological options: The non-pharmacological options for preventing PTSD are psychotherapy, physical activity, ICU diaries, comprehensive treatments, and health education [30]. ICU diaries are an intervention that is being studied for their potential to lower ICU-related PTSD, anxiety, and depression. A recent study demonstrated that ICU diaries can be beneficial if they are utilized to support the work within a program providing wrap-around services and close psychiatric follow-up for patients [31]. In their study, Jones et al. concluded that the provision of ICU diaries is an effective way of reducing the incidence of new PTSD and aiding in the psychological recovery of ICU patients [32].

The cognitive model of Ehlers and Clark for PTSD formed the basis of cognitive therapy for PTSD [33]. Cognitive therapy was indicated as the first line of treatment for PTSD by the National Institute for Health and Care Excellence [34]. Previously, various studies have shown that cognitive therapy is an effective treatment option for PTSD [35]. The non-psychological interventions are also of great importance. Strategies for restoring physiological sleep patterns, optimizing analgesia, reducing delirium, and sedation should be practiced in the ICU. Physiological intervention can also be beneficial psychologically and need for more complex and personalized treatments [29]. The non-pharmacological interventions are listed in Table 1.

**Table 1:** Various non-pharmacological options for the treatment of ICU-related PTSD ICU: intensive care unit; PTSD: post-traumatic stress disorder Reference: [36]

Non-pharmacological intervention	Methods
Psychotherapy	Through psychoeducation
Therapy with elements of nature	Use animal-assisted or horticultural therapy
Mindfulness therapy	Sessions of meditation, body and emotional perception, and awareness
Physical exercises	Sports, yoga, stretching combined with mental relaxation exercises
Art therapy	Use elements of psychology and arts (paintings)
Diet supplement therapy	Omega-3 capsules

Pharmacological options: Pharmacological interventions have shown promising results in lowering the severity of symptoms of PTSD; however, the overall impact of medications is uncertain. A meta-analysis supported the effectiveness of selective serotonin reuptake inhibitors (SSRIs) in PTSD. Commonly used SSRIs include sertraline, fluoxetine, and paroxetine along with serotonin-norepinephrine reuptake inhibitor (SNRI) venlafaxine and the atypical antipsychotic quetiapine as a stand-alone treatment for PTSD. Sertraline, a selective SSRI, was identified as an FPA-approved treatment for PTSD in this review. On meta-analysis of the randomized controlled trial, approximately 50-60% response rate was revealed in patients receiving sertraline therapy [37,38]. A brief overview of the different classes of drugs that are commonly used for PTSD treatment, their examples, and their mechanism of action are given in Table 2.

**Table 2:** Commonly used PTSD drugs GABA: gamma-aminobutyric acid; SNRI: serotonin and norepinephrine reuptake inhibitors; SSRI: selective serotonin reuptake inhibitors; PTSD: post-traumatic stress disorder

Classes of drugs	Use	Example	Mechanism of action	References
Antipsychotics	Used to improve sleep symptoms	Quetiapine, risperidone	Serotonin-5-HT_2_ and dopamine D_2_ antagonism	[39,40]
Sedative hypnotics or anxiolytics	Sleep symptoms	Prazosin	α_1 _adrenoreceptor antagonism	[41]
	Not efficient	Clonazepam, alprazolam	GABA_A _receptor antagonism	[42,43]
Anti-depressants	Used to improve overall PTSD symptoms	Mirtazapine	Adrenoreceptor α_2_ and serotonin-5-HT_2_ antagonism	[44]
	Used to improve overall PTSD symptoms but not efficient in managing heightened arousal symptoms	Venlafaxine	SNRI	[45]
	Overall symptom improvement	Paroxetine	SSRI	[46]
		Fluoxetine		
		Sertraline		

Role of ICU Diaries in Managing PTSD

It has been thought that ICU diary interventions can somewhat lower the symptoms of PTSD. Following are the randomized controlled trials of the past five years that were aimed at utilizing the ICU diary intervention to control PTSD in ICU patients (Table 3).

**Table 3:** Characteristics of the randomized controlled trials found in the literature ICU: intensive care unit; IES-R: Impact of Event Scale-Revised; HADS: Hospital Anxiety and Depression Scale; PTSS-14: Post-Traumatic Stress Syndrome; PTSD: post-traumatic stress disorder

Study and year	Country	Participants	No. of participants	Tool of evaluation	Follow-up	Outcomes	Findings
Rashidi et al. (2024) [47]	-	ICU patients	30	IES-R ICU-MT	3 months	PTSD score in the intervention was low (P>0.0001)	The nurse-initiated diary was effective on overall PTSD score and recall of patient memories
Torres et al. (2020) [48]	-	ICU patients	134	-	-	Participants who received diary intervention had a lower incidence of PTSD symptoms	Diary writing intervention during hospital stay and after discharge can lower PTSD in ICU patients
Wang et al. (2020) [49]	China	ICU patients	83	HADS IES-R	3 months	PTSD symptoms were observed, and ICU diaries were not useful in preventing them	ICU diaries can prevent hyperarousal symptoms and improve sleep quality and factual memory but could not lower PTSD symptoms
Nielsen et al. (2020) [50]	Denmark	ICU patients and relatives	75	PTSS-14 HADS	3 months	PTSD was observed and lowered in the diary group. There was no difference in anxiety and depression	ICU diaries written by the relatives of the ICU patients can reduce PTSD risk but were not positively effective in improving anxiety, depression, and quality of life
Garrouste-Orgeas et al. (2019) [51]	France	ICU patients	657	IES-R HADS	3 months	PTSD symptoms were not resolved with the use of ICU diaries	The findings of this study did not support the use of ICU diaries to lower or prevent ICU PTSD

Future directions and research gaps

There is considerable progress in understanding ICU-related PTSD, but there are several areas that require further exploration to enhance our understanding of this debilitating condition. One of the main future directions is to deepen our understanding of the underlying mechanism of ICU-related PTSD. Further investigations are required to explore the neurobiological changes that occur in the brains of individuals with ICU-related PTSD, as well as the impact of psychological factors such as coping mechanisms and social support. Early identification and prevention are another area that requires further investigation. Currently, there is a dearth of knowledge regarding the long-term outcomes and quality of life for persons who develop ICU-related PTSD. Therefore, the effectiveness of different treatment approaches for ICU-related PTSD needs further exploration.

## Conclusions

ICU-related PTSD is a significant and predominant issue among patients who have been admitted to the ICU. There are several screening tools for the identification of symptoms of PTSD. The most common symptoms include intrusive memories, anxiety, flashbacks, and nightmares which can greatly impact their quality of life and overall mental well-being. By offering psychological interventions, healthcare providers can help mitigate the long-term consequences of ICU-related trauma. However, there is a need for more improved healthcare practices in the management of ICU-related PTSD. Further studies are required to better understand the risk factors, prevention strategies, and optimal treatments for this condition.

## Appendices

Appendix 1

**Table 4:** Search strategy for the review TI: title; AB: abstract; CINAHL: Cumulative Index to Nursing and Allied Health Literature

Key variable	Subterms	Search options	PubMed	CINAHL	Web of Science
ICU	Intensive care unit	TI OR AB	139,161	65561	152,013
	ICU	TI OR AB	89,859	40354	86,113
	Critical care	TI OR AB	45,710	33700	104,794
	Critically ill	TI OR AB	62,975	27830	60,279
	Critical care ward	TI OR AB	30	240	1965
	Intensive treatment unit	TI OR AB	74	1096	33,002
	Emergency care unit	TI OR AB	248	1912	17,334
Total 1			244,168	126461	316,485
PTSD	PTSD	TI/AB	35,393	14631	38,733
	Post-traumatic stress disorder	TI/AB	17,446	6797	19,086
	Post-traumatic neuroses	TI/AB	15	0	83
Total 2			41,450	17533	46,456
Overall total = 1 and 2			773	425	962
